# Decision-making experiences of patients with end-stage kidney disease (ESKD) regarding treatment in Ghana: a qualitative study

**DOI:** 10.1186/s12882-018-1175-z

**Published:** 2018-12-19

**Authors:** Edward Appiah Boateng, Linda East, Catrin Evans

**Affiliations:** 1 Department of Nursing, Kwame Nkrumah University of Science and Technology, Kumasi, Ghana; 2 School of Health Sciences, University of Nottingham, Nottingham, UK

**Keywords:** Patient decision-making, End-stage kidney disease, ESKD treatment, Renal replacement therapy, Ghana

## Abstract

**Background:**

This is the first qualitative study to explore patient decision-making regarding end-stage kidney disease (ESKD) treatment in sub-Saharan Africa. The study addresses an important gap in the literature concerning choice and decision-making in an international context.

**Methods:**

The study employed a qualitative research design, using grounded theory methodology. In-depth interviews were conducted with twenty-two adult patients with ESKD in 3 clinical settings in Ghana. Data analysis involved coding and a constant comparative approach to generate key themes. Ethical approval was gained from relevant ethics committees both in Ghana and the United Kingdom.

**Results:**

Four main factors (personal, financial, healthcare system, and support network) were identified to influence patient decision-making regarding ESKD treatment in Ghana. Treatment was initiated for various reasons, including, initially, the urgent need to avoid premature death. Many approached their condition hoping for a cure and did not always understand the chronic nature of their condition. Financial and geographical inaccessibility of renal replacement therapy (RRT), as well as a relative lack of biomedical treatment choices, made decision making daunting for the individual with ESKD in Ghana. The subject of death or conservative management was not openly discussed. Rather patients did everything possible to seek alternative forms of treatment, including the simultaneous use of other non-RRT and traditional or faith-based healing approaches.

**Conclusions:**

Whilst similarities exist, this study illuminates stark cultural and contextual differences which make decision-making on ESKD treatment a daunting experience for the individual with ESKD in Ghana - as compared to those in high-income countries. The challenges associated with ESKD management in Ghana calls for meticulous efforts at primary prevention of the disease, including interventions directed at effective management of diabetes mellitus, hypertension and other chronic kidney disease (CKD) precursor conditions. Enhancing information provision would promote informed decision making, particularly within the initial stages of patient decision-making.

**Electronic supplementary material:**

The online version of this article (10.1186/s12882-018-1175-z) contains supplementary material, which is available to authorized users.

## Background

Individuals with end-stage kidney disease (ESKD) have to come to terms with a life-threatening condition, deal with inadequate information, select from limited choices, and carefully evaluate alternatives during decision-making regarding their treatment [1]. Consequently, patient decision-making in the management of ESKD has received increased attention [1, 2]. Several systematic reviews indicate that patients with ESKD draw upon their personal values and make judgements about the potential impact of treatment on their quality of life as well as survival advantage when making decisions about renal replacement therapies (RRT). Opinions of family members and/or clinicians, knowledge of other patients’ experiences, self-perceived burden to others, and desire to maintain lifestyle are among reported factors that inform decision-making among patients with ESKD [1–4].

The research on patient decision-making in ESKD, however, has come mainly from high-income countries, particularly from North America and Western Europe. This is despite the fact that the incidence of ESKD in low- and middle-income countries (LMICs) is greater than that of high-income countries, occurring mostly in a relatively younger age group (between 20 and 50 years old), with many of the affected individuals being breadwinners of their families [5–9]. It is estimated that around 70% of the global incidence of ESKD will be within LMICs by the year 2030 [10–12]. Generally, patients with ESKD represent around 0.03% of the total population in many high-income countries but their treatment alone takes about 2 to 3% of the annual healthcare budget [13]. Meanwhile, the higher costs of managing ESKD hugely impedes initiation and sustenance of RRT in many LMICs [12, 14–16].

To date, there have been no studies on treatment decision-making in ESKD conducted in the LMICs of sub-Saharan Africa. Understanding the issues faced by patients when diagnosed with ESKD in a healthcare setting with limited treatment options is critical to addressing their deepest concerns and influencing health system reform. This paper reports part of a larger study which led to the development of a grounded theory of ‘reconstructing health expectations’. The aim of this specific paper is to describe decision-making experiences of patients with ESKD regarding treatment in Ghana – and in doing so, compare and contrast with those reported in the wider international literature [1–4]. As such, it offers a unique contribution to the global literature on patient decision-making as to how patients with ESKD make decisions about their treatment in LMICs. Other aspects, including the grounded theory, will be reported elsewhere.

## Methods

### Research setting

This study was conducted in Ghana where financial, geographical and infrastructural barriers hinder efficient renal services, a situation common to most sub-Saharan African countries [16]. A 2010 census undertaken by the Ghana Statistical Service [17] estimated the total population of Ghana at 24,658,823 (48.8% male and 51.2% female). This represents a 30.4% increase from the total population in 2000 (18,912,079). Life expectancy is 63.4 years at birth. Ghana is classified as a lower-middle-income country by The World Bank [18] and ranks 15th among 48 countries in sub-Saharan Africa with a GNI per capita of $1590.

There is no national registry for chronic kidney disease in Ghana although efforts are being made to establish one, in association with other African countries [19]. Prevalence of CKD among persons with hypertension in Ghana is estimated to be 46.9%, and this increases to about 55% amongst those with pre-existing diabetes mellitus [20]. However, obtaining precise data on the prevalence of CKD is hampered by the fact that so many patients with CKD in rural or poorer areas go undiagnosed due to limited expertise and service availability.

In order to build a comprehensive picture of renal care in Ghana for this study, contacts were made with nurse leaders in all renal centres in the country to describe their patient and treatment characteristics. Details of their responses are provided in Table 1.

**Table 1:** Details of renal centres in Ghana (Cost estimated using exchange rate of USD 1: GHS 4.5, prevailing rate at the time of preparing the manuscript; HD – haemodialysis)

	Unit	Number of dialysis patients			Age Range	Average number of Sessions per Week	Number of HD stations	Cost per session (USD)
		Males	Females	Total				
1	Komfo Anokye Teaching Hospital^a^	5	2	7	26–78	2	5	42
2	NAHGE^b^	12	2	14	19–76	2	8	44
3	Police Hospital^a^	16	2	18	23–79	2	6	56
4	Ghana-Canada Medical Center^b^	3	3	6	26–50	3	3	56
5	Korle Bu Teaching Hospital Renal^a^	160	98	258	11–82	2	17	58
6	Korle Bu Teaching Hospital Cardiothoracic Centre^a^	21	17	38	27–85	3	10	58
7	Peace and Love Hospital^b^	5	2	7	38–67	2	4	47
8	Cape Coast Teaching Hospital^a^	26	11	37	26–68	2	10	56
9	Accra Kidney^b^	5	4	9	35–60	2	6	58
10	Benghazi Dialysis Unit^b^	9	1	10	33–50	2	5	56
Total		262	142	404			74	

^a^– government-funded facility; ^b^ – private-funded facility

Haemodialysis and, in a few cases, kidney transplantation with expatriate support, are the only available RRT modalities in the country. Haemodialysis services are provided by either government-funded or private-funded renal centres, with mainly nurses, medical officers and a limited number of nephrologists providing services within these facilities. The centres generally have an adequate number of nurses although they are mainly trained on-the-job. There are inadequate resources to meet the increasing demand for haemodialysis. For kidney transplantation, transplant surgeons and nurses from abroad travel to Ghana to perform the surgeries with a team of nephrologists and nurses at the Korle Bu Teaching Hospital Renal Centre. Patients and donors (live donors in all cases so far) complete all required preparations before the team from abroad arrives.

There were only ten renal centres in the country at the time of data collection, with all ten located in four major urban areas within the country and leaving much of the country with no specialist renal services. In some instances, these renal centres were established as a result of philanthropic acts, with no on-going plans for long-term sustainability. The seventy-four haemodialysis stations nationwide serve a population of about 25 million people, compared with a mean of 90 haemodialysis stations per million population in European Union countries [21], highlighting the inadequacy of service provision (peritoneal dialysis is currently not available for adult patients with ESKD due to the logistical challenges of importing the necessary dialysate solutions). Though very few, these renal centres utilise standard procedures and practices including using arteriovenous fistulas for those who are adequately prepared for haemodialysis and using disposable lines and dialysers. Ghana has a comprehensive national health insurance policy to promote universal health coverage and this initiative is being applauded internationally [22–25]. However, the higher costs of haemodialysis have led to its exclusion from the menu of treatments funded through the National Health Insurance Scheme (NHIS), even for the management of acute kidney injury, which was previously covered. As a result, individuals have to make out-of-pocket payments for treatment averaging $540 per month. One haemodialysis session costs around $45 while the net income of the average Ghanaian is about $250 per month. Consequently, few individuals with ESKD are able to afford treatment, often opting for two rather than the recommended three sessions per week.

### Study design and data collection

This study employed a qualitative research design, using grounded theory methodology [26–28]. Data was collected from the renal centres of two major teaching hospitals and a private hospital in 2014. These centres were selected because they are relatively well established and had all been operational for more than five years at the time of data collection.

Twenty-two adult patients with ESKD were selected using a theoretical sampling approach and interviewed for this study. Theoretical sampling required that, after initial purposive sampling, further sampling decisions including characteristics of participants, sample size, and data to be collected were made with reference to emerging categories [26, 27, 29]. Eligible participants were approached first by a nurse working in the respective renal centre, then by the first author, EAB, to discuss goals of the research. Five eligible participants declined participation because they did not feel well enough for the interview. Those who agreed to participate signed or thumb-printed the consent form and were subsequently interviewed by EAB. Five patients on haemodialysis, irrespective of their duration on the treatment, were identified, recruited and interviewed initially through the use of purposive sampling [26–28]. These initial interviews were transcribed and analysed for emerging concepts and categories. Memos were written on these initial categories and used to explore new ways to gather more data, and from which group of eligible participants. Indeed, memo-writing is a key process in grounded theory research as it prompts the researcher to analyse ideas about data, codes and categories in several possible ways [28]. This makes the researcher more analytic and reflective while coding, and aids retention of and elaboration on insights that aid development of theoretical codes [30]. Following this initial analysis, a decision was made to recruit and interview individuals who had been on haemodialysis for at least 12 months. The aim was to explore their experiences over the period that they had been on treatment. Five individuals who met this criterion were identified, with support from clinicians in the various renal centres, and interviewed.

Eight patients who were yet to start haemodialysis and one patient who was about to undergo kidney transplant surgery were then interviewed. The latter had been on haemodialysis for 12 months. The aim was to explore experiences of patients with ESKD who were at the stage of starting renal replacement therapy. An individual who had undergone kidney transplant surgery was also interviewed. More memos were written alongside concurrent data collection and analysis [28, 31]. The data suggested that it would be useful to interview individuals who had made decisions not to be on haemodialysis or those who had withdrawn from haemodialysis. The aim was to explore their health expectations at a stage where having a renal replacement therapy was less likely. However, no person meeting these criteria was identified as both groups mainly terminated their visits to the renal centres. However, two individuals who were considering quitting haemodialysis were identified and interviewed.

Data was collected through semi-structured, one-to-one interviews, with no other person present during the interviews. A semi-structured interview guide (Additional file 1) was developed in line with the objectives of this study. The first interview was to serve as a pilot interview but was included in the data analysis since the guide did not require major changes after this interview session. Interviews were conducted in the English language (15) or Twi (7), a popular Ghanaian dialect and the first language of EAB, based on the preference of each participant. All participants preferred to be interviewed in their respective renal centres. Each interview lasted for 30 min or more, with the longest lasting for 63 min. No repeat interviews were carried out.

Ideally, theoretical sampling should continue until saturation is achieved. This is when concepts and categories have been adequately well-defined such that collecting additional data cannot expand the properties of the category and variations and links between categories can be clearly explicated [26, 29, 32]. However, researchers may have to acknowledge a point at which concepts have been adequately explored and well-defined for the purposes of that particular research and report on areas that have not been explored as part of the limitations of the study [27]. Regular discussions were held among all researchers involved in this study on emerging categories and those that needed further exploration were agreed upon. Data collection continued until all researchers came to an agreement that key concepts had been adequately defined and explored for the purposes of this study. As explained earlier, not all concepts could be fully developed at this stage because of challenges in accessing participants that could provide the data to refine them. The reviewed literature showed that involving family members as well as informal care givers of individuals with ESKD provides different perspectives on the patient decision making process [2, 3]. However, as the focus of this study was on the subjective experience of individuals with ESKD, the decision was made not to include the perspectives of family members and informal caregivers at this stage.

### Data analysis

All interview sessions were audio-recorded and then transcribed. EAB translated the seven transcripts in the Twi language to the English language, and two independent researchers who are fluent in both Twi and English languages verified the accuracy of the translation. Each interview transcript was read by EAB while listening to the audio version to verify its accuracy, after which coding began. No software was used for the analysis of data from this study. This was to allow EAB to gain mastery of manual qualitative data analysis as the availability of qualitative data analysis software such as NVIVO may not always be guaranteed in his setting [33]. Coding involved three stages in this study. The first stage comprised conceptualisation and categorisation of data (open coding), followed by identification of patterns and relationships between categories (axial coding) and, finally, identification of an overarching category and linking it to the other categories (selective coding) [27, 34]. Data from different participants were constantly compared to identify similarities as well as differences during analysis. Emerging categories were also compared and contrasted in order to delimit their definitions and properties [29].

### Ethical considerations

Ethical approval was obtained from the Committee on Human Research, Publications and Ethics (CHRPE, KNUST) and the Ethical and Protocol Review Committee (EPRC, University of Ghana Medical School), both in Ghana, and from the Medical School Research Ethics Committee, University of Nottingham, United Kingdom.

Participating in an interview regarding a serious illness could potentially be emotionally difficult for some of the participants. They were assured that they could choose not to respond to a question if they judged it uncomfortable or private. Eligible participants were also assured that participation in the research was entirely voluntary and they were not under any obligation to participate. Once they agreed to participate, an informed consent form was given for them to sign or thumbprint to buttress their verbal consent before being interviewed. Participants were assured of confidentiality. Names and other personal identifiers were replaced by pseudonyms to ensure anonymity.

### Reflexivity within the research process

Reflexivity describes a process whereby a qualitative researcher performs a critical self-reflection to ascertain how his or her personal values, assumptions, decisions, and interpretations could influence the research [27, 28, 35, 36]. As such, reflexivity increases transparency and trustworthiness of qualitative research process and its outcomes [37, 38]. EAB is a nurse lecturer from Ghana and LE and CE are British nurses and academics in a UK university. EAB and LE both had previous experience of working with patients with ESKD. EAB undertook an MSc in the UK and the choice of the research topic arose from discussions between EAB and LE on the ethics of care and differences of experience for ESKD patients in Ghana and the UK. EAB then took this interest forward into a PhD study. EAB and LE both visited the three renal centres that were used for the study while planning and developing the study design, all financed by a grant from the Wellcome Trust in 2013. An affinity with constructivism influenced the choice of methodology as the team’s ongoing dialogue revealed the many cultural differences in the ways in which care is organised, how patients respond and what illness means across countries.

## Results

### Characteristics of participants

Seventeen of the twenty-two participants were male while the remaining five were female. Indeed, this was a reflection of the ESKD patient population at the time of data collection, as shown in Table 1. The ages of the participants ranged from 24 years to 73 years. Only three were above 60 years old, the age for retirement from public service in Ghana. Meanwhile, of those under 60, nine were in full-time employment, nine were unemployed and one was a full-time university student. Unemployment is a general problem within the country, with many of the participants being unemployed even before being diagnosed. The burden of treatment takes a great toll on an individual’s employment chances unless they are self-employed or there are special arrangements with their employers to support them, including flexible working hours, in order to meet their financial obligations and to remain in employment.

Fourteen participants were receiving RRT (13 haemodialysis and one kidney transplant) while eight had not started any form of RRT at the time of their interviews. Participants receiving RRT had been on treatment for periods ranging from 1 month to 11 years – ten of the 14 had been receiving RRT for at least 12 months, while only five of the 14 had been receiving RRT for more than 2 years. Four of the 22 participants were receiving some form of financial sponsorship from their employers for treatment while the remaining 18 were making out-of-pocket payments. Table 2 summarises key characteristics of participants, with all names changed to protect confidentiality.

**Table 2:** Characteristics of participants

	Participant	^a^RRT status	Duration of Haemodialysis/Diagnosis	Weekly sessions	Payment for treatment	Employment	Other known chronic condition(s)
1	Agya	HD	9 months	2	Out-of-pocket	Retired	None
2	Obrempong	HD	12 months	3	Out-of-pocket	Full time/ part-time Student	Hypertension
3	Barima	HD	1 month	2	Out-of-pocket	Student	None
4	Kantinka	HD	15 months	2	Out-of-pocket	Unemployed	Hypertension
5	Nana	HD	6 months	3	Sponsored	Retired/Part-time teaching	Hypertension, diabetes mellitus
6	Kwasi	HD	5 years	2	Out-of-pocket	Unemployed	None
7	Onua	HD	6 years	2	Sponsored	Full time (healthcare)	Hypertension
8	Wofa	HD	11 years	3	Sponsored	Retired	Hypertension
9	Adwoa	HD	19 months	2	Sponsored	Full time (teaching)	None
10	Abena	HD	2 months	3	Out-of-pocket	Full time (self-employed)	Diabetes
11	Akua	HD	4 years	2	Out-of-pocket (Support from Church and Rev. Minister)	Full time (teaching)	Hypertension
12	Eno	HD	12 months	2	Out-of-pocket	Unemployed (quit trading)	None
13	Owoahene	HD	12 months (3 days away from transplant)	2	Out-of-pocket	Unemployed	None
14	^b^Papa	T	16 months (2 years transplant)	3	Out-of-pocket	Full time (self-employed)	Hypertension
15	Akonta	N	21 months	N/A	Out-of-pocket	Employed	Sickle cell disease
16	Kwadwo	N	3 years	N/A	Out-of-pocket	Unemployed	Hypertension
17	Kwabena	N	1 year	N/A	Out-of-pocket	Unemployed	None
18	Kwaku	N	4 months	N/A	Out-of-pocket	Unemployed (quit driving)	Hypertension
19	Yaw	N	1 year	N/A	Out-of-pocket	Unemployed (quit driving)	None
20	Kofi	N	3 months	N/A	Out-of-pocket	Employed	Hypertension
21	Kwame	N	3 years	N/A	Out-of-pocket	Unemployed	None
22	Piesie	N	2 years	N/A	Out-of-pocket	Self-employed (trader)	Hypertension

^a^RRT Status – HD: Haemodialysis; T: kidney transplant; N: Non-dialysis

^b^Participant started haemodialysis before having transplant

### Factors influencing patient decision-making

**Fig. 1:**
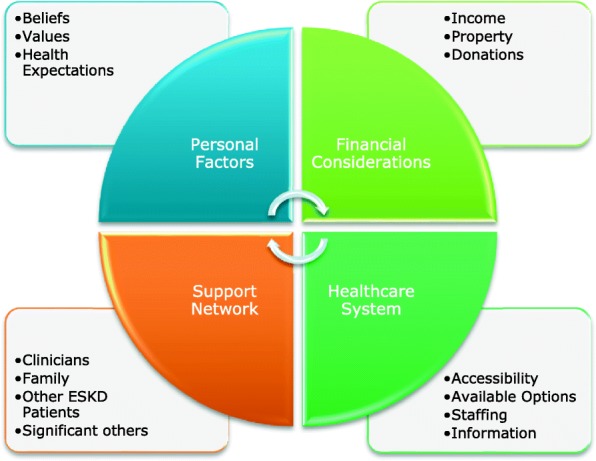
Factors influencing Patient Decision-making in ESKD in Ghana

This study found that patient decision-making regarding ESKD treatment in Ghana was influenced by several overlapping and interlinked factors. These are clustered within four main categories – (i) personal factors, (ii) financial considerations, (iii) healthcare system and, (iv) support network. Figure 1 shows these categories and their key components. The categories are explained further in the subsequent sections.

#### Personal factors

Following their diagnosis, participants drew upon their personal values and belief systems (including religious beliefs) to make sense of what was happening to them and in consideration of their subsequent decisions regarding treatment. Initially, almost all participants expressed a strong drive to start treatment and were often determined to do so (even where clinicians were more cautious), knowing that their lives were at stake without haemodialysis.

*“Then he* [the doctor] *said ‘oh, then when we are going to treat you it will cost you much money so it’s better, the money that you will use for the treatment, it’s better you go home and then use the money to look after your children.’ So I was thinking ‘ah, then does it mean I have to die because of money or what? So I told him no, I can look after myself so he should rather treat me” (Agya, HD).*

*“Initially, I knew there was no option, either I do it* [haemodialysis] *to survive or I die” (Obrempong, HD)*

Knowing that their disease is potentially life-threatening, participants felt that starting treatment was a way of saving themselves for that moment, whilst hoping for better potential outcomes in the future, often influenced by their religious convictions.

*“What about if I decide to leave myself the way I was* [without treatment] *and then, possibly, something happens to me? I wouldn’t know what nature or what God has in store for me the following day or two. So I was just trying to save myself for the moment, believing that tomorrow will be better” (Owoahene, HD).*

“I believe that for any illness that we have in this world … God has created a way out of such situations and it is because of ignorance that we have not been able to get there. So I believe that there is some treatment outside dialysis which mankind may discover sooner or later” (Wofa, HD)*.*

Related to the above point, a key finding was that participants did not always understand the chronic nature of their disease at the time of initiating treatment. Many started treatment believing there was a cure ahead.

“I didn’t know it was going to take a long time like this…yeah, I felt within 6 months I have to be cured” (Agya, HD)*.*

*“I knew that when* [I] *come to dialysis for, let’s say 10 times or 6 times, it will go. So I was coming here happy since I was going to be OK” (Barima, HD).*

Mistakenly optimistic expectations of ‘cure’ were largely influenced by inadequate information and spiritual and religious beliefs that promise a cure for almost all kinds of diseases.

*“Some diseases are bought* [spiritually] *for you and the more you pray, although the disease would have been given to you already, the more you pray, the person can withdraw so that when you pray well God will help so that the sickness will go away” (Yaw, N).*

*“Well, we know our God, there is no disease or illness that He cannot cure and mine is not* [an] *exception…the medical situation that we have available cannot give me 100% treatment but God, I know God can, even transplant does not…solve all my problems, only some” (Owoahene, HD).*

In summary, although participants appreciated the life-threatening nature of ESKD following their diagnosis, they drew on their values, beliefs (particularly religious beliefs) and normative understandings around disease trajectory (believing in a future cure) when faced with decisions regarding treatment. In fact, many hoped for a cure in the time period when they were making decisions about initiating and sustaining treatment. Participants did not generally represent ESKD as terminal illness and did not report having conversations with healthcare providers about the possibility of conservative management or end-of-life care. However, it is noteworthy that the drive to initiate treatment alone was insufficient for treatment initiation as other important factors had to be considered in the decision-making process. The next section presents financial considerations as a key factor that influences even how long a patient with ESKD in Ghana can be sustained on treatment.

#### Financial considerations

Dialysis costs are expensive, and for the individual who has to make out-of-pocket payments for treatment, the psychological trauma resulting from being diagnosed with life-threatening ESKD was worsened by thoughts about how to raise money to pay for treatment. The financial position of an individual or their significant others was, therefore, instrumental in decisions about haemodialysis initiation and sustenance.

“The only problem I have now is the cost. I see many patients who, apart from the clinical issue, are tormented by issues about finance” (Nana, HD)*.*

“Can you imagine, as a civil servant, how much do I earn? If I pay 450 Ghana cedis (about $100) a week, not to talk about the other injections…how many patients can afford that? It's terrible” (Obrempong, HD)*.*

Some participants reported having to use their personal savings to start haemodialysis while others relied on donations from relatives or employers to pay for their treatment. Some had plans to sell their property in order to sustain treatment.

*“What I will do is to sell some of them* [property] *and put it in a sinking fund that will run itself so monies will be generated from those investments to take care of it” (Obrempong, HD).*

“I had saved from my trading, so I used that initially. I also got financial support from my younger sister. But I have spent all my savings now and my sister is also having financial challenges” (Eno, HD)*.*

“*Even with these* [erythropoietin] *injections I get financial support from my friend, without him I couldn’t have started it. He is abroad so whenever I am in need of money he sends me some. Even he brought me the money I used to purchase the ones I have now” (Kwadwo, N).*

Consequently, only a few participants could afford the recommended three haemodialysis sessions per week. A key concern for many participants, therefore, was the fear of dying due to lack of funds to pay for continuing treatment. Treatment discontinuation was not mentioned as an option in relation to palliative care choices, but rather as something that would only occur if patients ran out of money – hence it was not planned or supported as a ‘decision’ as such. Thus, participants had to consider their financial situation when making decisions about initiating treatment, and this could determine how long and how often treatment would be. Yet, the study also identified situations where individuals could pay for their treatment but could not get their desired choice, owing to the healthcare setup in the country. The next section presents how the healthcare system influenced the decision-making of patients with ESKD in the country.

#### Healthcare system

For many participants, treatment choices were strongly shaped by the (limited) geographical availability of treatment centres. In fact, the only realistic option for some was either to relocate or travel long distances across regions for renal services. However, the latter was impractical considering how many times haemodialysis patients, for instance, have to frequent a renal centre. Relocating to another region also presented its own challenges, including the potential loss of employment for those who were not self-employed. Under such circumstances, individuals were more likely to abandon biomedicine in search of other alternatives.

*“Since I’m working I can’t have that time coming three times in a week, from* [Eastern Region] *to this place* [Greater Accra Region]*. It will be a bit hectic … I don’t know which work will give me that excuse duty to be coming for treatment three times in a week. Three days off? No, it’s not possible” (Akonta, N).*

Another key influence on treatment decision-making of the Ghanaian patient with ESKD is in relation to the available modalities. Haemodialysis is the only modality that is consistently available in the country, prompting some to seek their preferred treatment overseas.

*“We were schooled that the ultimate* [treatment] *is transplant … … somebody went to India and was encouraging me that they have a lot of donors … … so the little money I had, having sold some properties, I decided to go to India” (Obrempong, HD).*

The pluralistic nature of healthcare delivery in Ghana implies that individuals with ESKD may choose to seek healthcare through avenues other than biomedicine, including traditional medicine and faith healing, in line with their beliefs. Patients are generally advised against using these other approaches as there are no data supporting their effectiveness in managing ESKD. Yet, some participants reported having used some of these other approaches, sometimes alongside biomedicine.

*“Oh, I tried it* [using traditional medicine] *for some time, about 6 months. It wasn’t too bad … I was coming* [for dialysis] *and I was doing my tests” (Wofa, HD).*

“Recently, a customer of mine insisted that I go to church with him …sometimes, you just want to try. You are sick and you don’t know whether you will get better there. Also, there are times when the doctors say that ‘at this stage, all you can do is to pray’. So you get confused” (Yaw, N)*.*

Healthcare system also includes things like staffing, communication and information availability. Patients who are diagnosed with ESKD in Ghana face enormous challenges, with some participants reporting not knowing anything about ESKD or its management before their diagnosis. Indeed, the role of clinicians as educators is critical in supporting these patients to address their informational needs while making decisions regarding treatment, although the quality of this input was variable.

“We come here every month...each time we come here they educate us about so many things including our diet… I feel that it is good they educate us when we come here” (Yaw, N)*.*

*“The education is low … I was here* [renal centre] *for almost two years but I didn’t have any idea about what is affecting me … .and there is some secrecy surrounding the whole thing. Because of the enormity of the financial commitment, they tend not to shock you with the information as to the nature of the disease so before you become aware, you become traumatised” (Wofa, HD).*

In summary, while the individual diagnosed with ESKD in Ghana hopes for a better tomorrow while making decisions regarding treatment, the healthcare system does not always nurture this hope, leading to diverse and challenging decision-making experiences.

#### Support network

Decision making on treatment is not a one-off process in ESKD but is ongoing. Confronting the realities of living with, and managing, ESKD, as well as taking steps to adjust to a ‘new reality’ means that patients with ESKD have to review their decisions each time they are due for treatment. Availability of a good support network facilitated decisions to continue ESKD treatment in diverse ways. Participants described the emotional and psychosocial support that they were getting from clinicians.

“I think the nurses here have been wonderful … I told you I broke down in this room, I cried. But…they spoke to me and they calmed me down a little bit” (Abena, HD)*.*

*“They* [clinicians] *will talk to you, they will call you on the weekends, any complications, any this and, it's so nice. Sometimes when they are having parties and other things they invite you to come to socialise and, it's so fun” (Obrempong, HD).*

An additional form of support that participants described is that from their family and friends.

*“And luckily, my* [kidney] *donor was a very young person, my niece, who was physically fit, in her late 20s and she didn’t have any other medical problem so it was quite OK” (Papa, T).*

*“Well, it was not so difficult* [getting a kidney donor] *because it is my brother that is giving me” (Owoahene, HD).*

Participants also described a support network that exists within the renal centres among patients with ESKD. This ranges from sharing information and ideas to, sometimes, financial support for other patients.

*“I talk to them* [other patients with ESKD]*, sit with them, I explain things to them. In fact, I even support a few of them if they are acutely financially distressed” (Nana, HD).*

This support network acts in many ways to reinforce the decision of ESKD patients to continue treatment.

In summary, an interplay between the above factors informs patient decisions regarding ESKD treatment initiation and sustenance in Ghana. While some are similar across all settings where decisions are made on ESKD treatment, others, such as financial considerations, healthcare system, and health expectations have aspects that are unique to LMICs, including Ghana.

## Discussion

The data presented above elucidates the patient decision making experience in Ghana and enables a tentative comparison to be made with that of patients in high-income contexts as reported in several systematic reviews [1–4] – see Table 3 for a summary of the similarities and differences.

**Table 3:** A summary of similarities and differences between the Ghanaian setting and that of many high-income countries

Similarities between Ghana and many high-income countries	Differences between Ghana and many high-income countries
1. Personal values/autonomy central to patient decisions 2. Support from clinicians, family and friends reinforce ongoing decisions about treatment	1. Decisions to initiate or sustain treatment are mostly driven by hope for a cure in the short-term, rather than an appreciation of the long-term management of the condition 2. Thoughts about how to raise money to finance out-of-pocket payment for dialysis is a concern for many patients in Ghana 3. The number of dialysis sessions is generally reduced to contain costs 4. Accessibility to and availability of RRT is an immediate concern for patients with ESKD in Ghana while those in many high-income countries usually do not have these concerns and may take service provision for granted 5. Palliative care for ESKD is not openly considered as a treatment option in Ghana, as is the case in many high-income countries

Individuals with ESKD in Ghana make decisions to start RRT (or not) based on their values, beliefs about disease trajectories and causation, availability of treatment modality as well as its affordability. These factors, while similar to those identified in existing systematic reviews in some cases [1–4], have different implications and meanings for the patient with ESKD in Ghana.

Patients with ESKD generally consider personal factors such as how treatment would affect their lifestyle, quality of life, and their perceived burden to other people when making treatment decisions [1–4]. Similarly, participants of this study were influenced by personal factors while making decisions about their treatment. Many were primarily making decisions with the ultimate purpose of preserving their lives, even where clinicians were expressing more caution. This highlights the autonomy of the individual with ESKD in making decisions about their treatment, even in resource-limited settings.

Whilst acknowledging the seriousness of their condition, many patients harboured hopes for, and expectations of, a cure. Such beliefs and expectations of cure were related to two factors – (i) their beliefs around disease causation (especially among those who attributed their illness to spiritual origins), and, (ii) their familiarity with communicable disease trajectories which are mostly curable. Patterns of diseases are changing globally, following Omran’s [39] theory of epidemiology of population change. While many LMICs are experiencing an epidemiological transition leading to growing rates of non-communicable diseases (NCDs), they also continue to experience a burden of communicable diseases whose management has historically shaped the major structures processes and practices of health systems and health education [40, 41]. Citizens of LMICs such as Ghana, therefore, are most familiar with the principles and policies used in managing communicable diseases (such as having a curative therapy that lasts for a short period). This study suggests that expectations of disease trajectory and cure around ESKD are still often shaped by this ‘curative’ paradigm rather than viewing ESKD as a long term and potentially terminal condition.

Patients in many high-income countries are maintained on RRT for as long as their health condition permits, or when a personal decision is made to withdraw from RRT. Yet, the lack of health insurance coverage for the treatment of ESKD means that patients with ESKD in Ghana have to continually take into account the affordability of treatment in their decision-making process. Indeed, many patients with ESKD in resource-constrained settings tend to be aware of the ongoing costs and short-term nature of RRT in resource-constrained settings [12]. While expensive for all, misguidedly optimistic expectations of a cure, hope for a better outcome of treatment, or the need to avoid premature death almost always led participants to use their personal savings or sell off their property to initiate and sustain treatment. Some initiated RRT with the intention of ‘buying time’ while pursuing other courses outside biomedicine that were in line with their beliefs such as faith healing and traditional medicine. While this may be because some could not afford to pay the higher cumulative costs of treatment by haemodialysis, others did this in search of a cure because they found it hard to accept ESKD as a disease without a cure.

There are suggestions that the quality of RRT in sub-Saharan Africa and other LMICs is suboptimal, especially among incident dialysis populations, contributing to poorer outcomes [10, 42]. Yet, Eghan et al. [43] partly attributed early mortality observed among incident haemodialysis patients in a renal centre in Ghana to financial constraints as they were unable to afford the costs of sustaining treatment. Adult patients with ESKD in such settings are relatively younger and usually have fewer comorbidities, and regular access to RRT tends to improve outcomes significantly, with over 75% surviving for 1 year and beyond [12, 16]. All participants in this study who had been on haemodialysis for 12 months and beyond had consistent access to haemodialysis, irrespective of whether they were having two or three haemodialysis sessions in a week. This suggests that consistent access to haemodialysis without financial hindrance could improve survival in patients with ESKD in Ghana and other LMICs, although this may need further exploration.

A key difference between patients with ESKD in Ghana and those in many high-income countries lies in the treatment choices requiring greater deliberation. For example, choosing “survival or death” is a decision generally requiring little deliberation for patients with ESKD in high-income countries. Furthermore, initiation or continuation of RRT might be seen as the default option because accessibility to RRT is not perceived as an immediate concern [44]. Instead, in a high-income context, the main decision is about RRT modality (haemodialysis or peritoneal dialysis in their various forms) [2, 3]. By contrast, the Ghanaian patient with ESKD who chooses survival does not need further deliberation on which treatment modality to choose as haemodialysis is the only obvious biomedical option in the country. Instead, the initial choice of ‘survival or death’ demands more deliberate decision making as they have to consider whether they can access haemodialysis, how to pay for their treatment, how long they can finance haemodialysis, and the frequency of haemodialysis sessions based on their finances. This means that while patients with ESKD in other settings have choices to suit their unique needs and lifestyle, lack of choice is acutely felt by the Ghanaian patient with ESKD who has the option of in-centre haemodialysis (albeit a few persons have home haemodialysis) and, rarely, kidney transplantation. Ghanaian patients with ESKD can choose how often to have haemodialysis sessions but this is largely dictated by their finances.

As has been stated earlier, the National Health Insurance Scheme does not provide coverage for the treatment of ESKD in Ghana. Rationing of treatment, in spite of inherent challenges, has been used in various forms in RRT since its inception [45, 46]. Efforts have been made to address some of these challenges in some LMICs, and there have been improved outcomes of RRT in African countries where governments totally or partially cover treatment costs for patients with ESKD, including Gabon, Mauritania and South Africa [42]. Indeed, in the face of limited resources, having clear-cut and transparent policies to guide allocation goes a long way to improve accessibility to RRT and outcomes [12] and could offer some relief to patients with ESKD in Ghana.

The prevailing circumstances in the country suggest that death is the ultimate outcome of ESKD and the option that will ultimately befall all patients with ESKD, whether sooner or later. Indeed, the fear of death is a similar drive across all settings when decisions about treatment of ESKD are being made. However, while palliative care/conservative management is often discussed as an option to managing ESKD in many high-income countries, this is not the case in the Ghanaian setting. In fact, there appears to be a degree of ‘secrecy’ or silence surrounding this as death is not discussed explicitly as a potential outcome of the disease. What is more, there are no organised palliative care services for ESKD patients within the biomedical setting. The Ghanaian patient with ESKD initiates treatment while nurturing hope for a cure. Patients interviewed for this study saw dialysis as an opportunity to buy time to search for a cure rather than as a palliative measure. In effect, although end-of-life care could be an option for the Ghanaian patient with ESKD, this study showed that it is currently unlikely to be discussed in depth or even mentioned.

Individuals with chronic diseases require support from family members and significant others to achieve effective self-management goals [47]. The support that patients with ESKD get, or its lack thereof, tends to reinforce their choices or make them consider other alternatives respectively. Indeed, the support network is highly instrumental in promoting the well-being of the individual diagnosed with ESKD. The existence of such emotional, psychological and practical support has been found to provide a context that allows individuals with ESKD to ‘be themselves’ while integrating their illness experience into their day-to-day lives [48]. Similarly, the existence of a good support network promotes initiation and reinforces the decision by individuals with ESKD to continue treatment of the disease in Ghana, although there is the urgent need to improve health information support for patients. Access to online information via the worldwide web is now available to most Ghanaians living in urban areas thanks to the spread of mobile technology. However, health literacy is limited, as is the information provided within health care services.

### Limitations

The views and opinions of family members could have added to the rich data presented in this study. This is the first study to explore patient decision-making in persons with ESKD in a sub-Saharan African country. Thus the focus was on understanding the subjective experiences and perceptions of those with the disease. Further studies exploring the perspectives of family members would provide a deeper understanding of how decisions to start treatment affect the entire family.

Data for this study came solely from patients with ESKD who had started dialysis, those who had undergone kidney transplantation, and those who were yet to start treatment. Views from patients with ESKD who had decided not to be on dialysis, those who had withdrawn from dialysis, or those who were using traditional medicine or pursuing faith-healing alone would have provided a different and very valuable perspective on decision making experiences. However, it was practically impossible to identify a patient who met these criteria in a typical renal centre. Patients with ESKD who withdrew from dialysis and those who chose not to start dialysis were either not followed up by the renal centre or were reported to have passed away. Getting their experiences would have provided an important alternative perspective.

Participants of this study comprised 17 males and only 5 females and could be misinterpreted as possible gender bias. However, as explained above, the gender differences noted was a reflection of the total dialysis population in the country at the time of data collection. It is known that access to healthcare for females is generally low, probably due to their relatively weaker economic and decision-making powers [15, 16, 49].

## Conclusions

This paper has explored decision-making experiences of patients with ESKD in Ghana regarding treatment and discussed similarities and differences between these and other settings. Personal factors in this study are similar to those identified in other studies on patient decision-making in ESKD in several instances. However, variations in underlying beliefs about illness causation in Ghana lead many to ‘buy time’ with treatment initiation while nurturing expectations of a cure. Utilising innovative ways of information provision about ESKD such as information leaflets, patient decision aids and the use of the media would enhance awareness about ESKD and its management. The financial aspect of decision making is entirely different in the Ghanaian setting as compared to many other high-income settings owing to the lack of health insurance policies to take care of costs of treatment. Financial constraints impact the length of time by which patients with ESKD are sustained by the only viable RRT option in the country and the concomitant use of other options outside biomedicine. The health system in Ghana presents options that are different from those presented to patients with ESKD in several high-income countries, causing the Ghanaian patient with ESKD to greatly experience restrained options and resulting in differences in choices made. Support networks identified in this study were described as similar to what exists for other patients with ESKD elsewhere.

Considering the numerous challenges faced by patients with ESKD in Ghana, as have been reported above, meticulous efforts directed at primary prevention of the disease, including effective management of diabetes mellitus, hypertension and other chronic kidney disease (CKD) precursor conditions would be the optimum approach. Finding innovative ways to reduce high costs associated with RRT will contribute to making RRT financially accessible to people who need it in low- and middle-income countries. Programmes such as ‘the affordable dialysis prize’ which was announced at the World Congress of Nephrology in Cape Town in March 2015 and awarded in March 2016 can bring ideas designed to lower costs of RRT to the fore [50]. Also, training of transplant surgeons in adequate numbers, coupled with promotion of live donor/altruistic kidney transplants and efficient follow-up care could reduce the cumulative costs associated with dialysis. Enhancing information provision would promote informed decision making, particularly within the initial stages of patient decision-making.

## Additional file

Additional file 1: Interview Guide: This guided data collection for this study. (PDF 509 kb)

## Abbreviations

CKD: Chronic Kidney Disease
ESKD: End-Stage Kidney Disease
HD: Haemodialysis
LMICs: Low- and Middle-Income Countries
N: Non-dialysis
RRT Status: Renal Replacement Therapy Modality for Patient
RRT: Renal Replacement Therapy
T: Kidney transplant
