# Supplementary material for: Decision-making experiences of patients with end-stage kidney disease (ESKD) regarding treatment in Ghana: a qualitative study
Source: BMC Nephrol. 2018 Dec 19;19:371. doi: 10.1186/s12882-018-1175-z (PMC6299918; doi:10.1186/s12882-018-1175-z)
Supplement: Supplementary file 1 — Interview Guide: This guided data collection for this study. (PDF 509 kb) [file 12882_2018_1175_MOESM1_ESM.pdf]

INTERVIEW GUIDE

1. *Please describe the history of your kidney disease to me*

Cues: when did you first find out that you were ill? What symptoms did you experience? What did you think was happening to you? What actions did you take to get well? When were you first told that you had kidney disease? How did you feel when you found out that you had kidney disease? What did you think might have caused your kidney disease? What made you think those factors might have caused your kidney disease?

2. *Please tell me what you knew about kidney diseases before your diagnosis?*

Cues: Had you heard anything about kidney disease? How did you get to know that? Did you know anybody with kidney disease?

3. *Can you tell me how you feel about your kidney disease?*

Cues: Do you feel it has affected your life in any way? Do you feel it is still affecting your life in any way?

4. *Please tell me any thoughts you have had about the treatment you are having?*

Cues: Can you tell what you know about the treatment? How did you get to know this? Do you know of any other treatments you could have had? How did you know about these? Would you rather you had any of the other treatment options?

5. *How did you decide to be on this treatment?*

Cues: Were you told about all possible treatments for you? Who told you about the treatment? Were you told about the treatment options alone or in the company of others (i.e. your spouse, children, other family members, other patients, employers)? Did you have much time to deliberate on what to do? Would you prefer to have received the information earlier or later? Why? Did these other people suggest what you should do to you? Did you talk to others with your diagnoses before deciding on the treatment? Were you made to choose the treatment you are on now? Do you think your interaction with all these people influenced your decision? What else do you think influenced your decision? What made you choose this treatment?

6. *How did you feel about deciding on a treatment?*

Cues: Did you feel valued? Did you feel the decision should have been taken by someone else other than you? Who should this person have been? Do you feel you were involved in the decision making to a greater extent?

7. *How do you feel about this treatment?*

Cues: What do you think are the good things about this treatment? What do you think are not so good about this treatment? How do you feel about the cost of the treatment? Do you feel this treatment is as helpful as you were told about it before you started? Is there something about the treatment that you feel you should have known before starting? Will this have made you refuse the treatment if you had known earlier? Do you intend to continue this treatment for long? Do you have any particular challenges with this treatment? Will you have chosen this same treatment if you knew all the things you now know about it when making the decision?

8. *Please tell me about your expectations while on this treatment?*

Cues: Do you expect a cure? Do you expect your health to deteriorate over time? Do you have any plans for how you should be cared for if your health deteriorates? Have you been told of what can be done for you should your health deteriorate?

9. *Do you have any ideas on how care for others with kidney disease can be improved?*

Cues: What do you think they should be told when they are diagnosed with kidney disease? How will you expect them to be treated? Who should make decisions for them on their treatment (the individual, the doctor, the spouse or family)? What could be done to help decision making? Is there anything else that might be helpful to others who have to make a similar decision as yours? How involved should they be in the decision making?

10. *Can you comment on the care that you have received in this unit?*

Cues: Have staff in the unit been supportive? How have they been doing this? Has the unit been running efficiently since you started coming here? Do you think care can be organised any differently to support others who have to make a decision?

11. *Do you have any general comments on what we have discussed so far?*
